# Mortuary Statistics of Natchez, Miss., for the Year Ending Dec. 31st, 1865

**Published:** 1866-03

**Authors:** J. Stebbins King

**Affiliations:** Surgeon in charge Mississippi State Hospital, Natchez, Miss.; late Recorder Board of Health


ARTICLE XI.

### MORTUARY STATISTICS OF NATCHEZ, MISS., FOR THE YEAR ENDING DECEMBER 31st, 1865.

Compiled By J. STEBBINS KING, M.D., Surgeon in charge Mississippi State Hospital, Natchez, Miss.; late Recorder Board of Health.

Editor Medical Examiner:—

I herewith transmit, for publication, a consolidated report oi deaths occurring in this city, for the year 1865. It is compiled from the records of the Board of Health, and the City Sexton's books.

The Board of Health was organized by order of Gen. Davidson, in the latter part of last January, and continued to keep the mortuary records until the 14th of September, when the City Sexton resumed his duties under civil rule.

The number of deaths from unknown causes appears large, for two reasons:—First, there was no complete record of diseases kept during the month of January; Second, the physicians of this city, as elsewhere, are not as particular as they should be, in regard to reporting the causes of death to the City Sexton.

I am now compiling the mortuary statistics of this city from the year 1850 to date, which, when completed, I will forward to you. I am also collating, as rapidly as possible, the mortuary statistics of all the principal cities and towns throughout the United States. And I would hereby request physicians to assist me, as far as possible, by forwarding me the mortuary reports of their several cities and towns, together with such topographical and meteorological accounts as may be of interest to the medical profession. As far as possible, I wish the reports to include the sixteen years from 1850 to 1865, inclusive. But where the mortuary reports are not complete for that length of time, forward such as are:—

| | WHITE. | | COLORED. | |
|---|---|---|---|---|
| | Male | Female | Male | Female |
| Apoplexy, | 2 | 1 | 2 | 1 |
| Abortion, | 0 | 0 | 0 | 1 |
| Angina Maligna, | 0 | 0 | 0 | 1 |
| Bronchitis, | 1 | 0 | 0 | 0 |
| Burned, | 0 | 0 | 0 | 1 |
| Birth, Premature, | 0 | 0 | 0 | 1 |
| Consumption, | 6 | 7 | 3 | 5 |
| Convulsions, | 2 | 1 | 1 | 4 |
| Congestion of Brain, | 1 | 3 | 0 | 0 |
| Cholera Infantum, | 1 | 3 | 4 | 0 |
| Colic, | 0 | 0 | 3 | 1 |
| Cancer of the Womb, | 0 | 0 | 0 | 1 |
| Croup, | 0 | 0 | 1 | 2 |
| Diarrhœa, Acute, | 5 | 1 | 13 | 14 |
| Diarrhœa, Chronic, | 3 | 1 | 1 | 1 |
| Dysentery, Acute, | 1 | 2 | 2 | 8 |
| Dropsy, | 1 | 2 | 2 | 4 |
| Dentition, | 2 | 0 | 4 | 2 |
| Drowned, | 1 | 0 | 1 | 0 |
| Debility, | 2 | 0 | 0 | 1 |
| Delirium Tremens, | 1 | 0 | 0 | 0 |
| Diphtheria, | 2 | 0 | 0 | 0 |
| Endocarditis, | 2 | 0 | 0 | 0 |
| Fever, Typhoid, | 2 | 1 | 2 | 4 |
| " Remittent, | 1 | 2 | 4 | 6 |
| " Congestive, | 2 | 1 | 0 | 1 |
| " Puerperal, | 0 | 1 | 0 | 0 |
| " Scarlet, | 0 | 1 | 0 | 0 |
| Gunshot Wound, (accidental) | 1 | 0 | 0 | 0 |

| | | | | |
|---|---|---|---|---|
| Hydrocephalus, | 0 | 0 | 0 | 1 |
| Injury from a Fall, | 0 | 0 | 1 | 0 |
| " of Spine, | 0 | 0 | 1 | 0 |
| Inflammation of Brain, | 2 | 0 | 0 | 2 |
| " Bowels, | 0 | 1 | 2 | 1 |
| " Heart, | 0 | 2 | 0 | 1 |
| " Liver, | 0 | 1 | 0 | 0 |
| " Womb, | 0 | 0 | 0 | 1 |
| Killed by Accident, | 0 | 1 | 1 | 0 |
| Mortification, | 1 | 0 | 0 | 0 |
| Measles, | 3 | 2 | 2 | 3 |
| Old Age, | 0 | 1 | 1 | 1 |
| Pneumonia, | 5 | 1 | 13 | 4 |
| Paralysis, | 1 | 1 | 0 | 1 |
| Rheumatism, Chronic, | 1 | 0 | 0 | 0 |
| Stillborn, | 7 | 1 | 9 | 2 |
| Small-Pox, | 0 | 1 | 0 | 1 |
| Tetanus, Traumatic, | 1 | 0 | 1 | 1 |
| Tabes Mesenterica, | 0 | 0 | 1 | 0 |
| Uterine Phlebitis, | 0 | 1 | 0 | 0 |
| Uræmia, | 0 | 0 | 0 | 1 |
| Unknown, | 15 | 12 | 23 | 29 |
| Whooping-Cough, | 0 | 1 | 0 | 0 |
| White Swelling, | 0 | 0 | 1 | 0 |
| Total, | 75 | 53 | 99 | 108 |
| Total of Whites, | | | 128 | |
| Total of Negroes, | | | 207 | |

Of the 90 whites who died previous to the 31st of July, only 20 were of families of old residents; the remaining 70 being almost entirely refugees, who came here from the swamps of Louisiana, many of them dying within 48 hours after their arrival in the city.

The deaths for the several months were as follows: January, 27; February, 30; March, 26; April, 24; May, 52; June, 44; July, 34; August, 23; September, 18; October, 25; November, 17; December, 15.

The ages were—

| | |
|---|---|
| Under 3 years, | 118 |
| Between 3 and 10, | 43 |
| " 10 and 25, | 43 |

| | | |
|---|---|---|
| " | 25 and 40, | 51 |
| " | 40 and 60, | 29 |
| " | 60 and 80, | 21 |
| " | 85, | 1 |
| Unknown, | | 29 |

The population of Natchez, as per census taken last spring, by the Board of Health, was:—

| | |
|---|---|
| Whites, | 3,558 |
| Negroes, | 5,026 |
| Total, | 8,574 |

Since that time, the white population has been considerably increased, and is probably at the present time not far from 5000; the colored population about the same. Thus Natchez now numbers about 10,000 inhabitants.

Estimating the average white population for the year to have been 4000, we would have a mortality of one to every 31⅛. And if we were permitted to deduct the 70 deaths of refugees, (who, properly, should not be added to our mortuary list, as, perhaps, without an exception, the diseases of which they died were contracted before arriving in the city,) we would have only one death to every 60 10-29 of the white population.

Estimating the average number of colored inhabitants for the year to have been 5000, we would have a mortality of one to every 24 32-207 of the colored population.

